# Air Backwash Efficiency on Organic Fouling of UF Membranes Applied to Shellfish Hatchery Effluents

**DOI:** 10.3390/membranes8030048

**Published:** 2018-07-23

**Authors:** Clémence Cordier, Christophe Stavrakakis, Patrick Sauvade, Franz Coelho, Philippe Moulin

**Affiliations:** 1 Aix Marseille Univ, CNRS, Centrale Marseille, M2P2 UMR 7340, Equipe Procédés Membranaires (EPM), Europôle de l’Arbois, BP80, Pavillon Laennec, Hall C, 13545 Aix en Provence CEDEX, France; clemence.cordier@univ-amu.fr; 2 Laboratoire Sécurisation des Productions en Conchyliculture, Station Ifremer de Bouin, Polder des Champs, 85230 Bouin, France; christophe.stavrakakis@ifremer.fr; 3 SUEZ–aquasource^®^, 20, avenue Didier Daurat, 31029 Toulouse CEDEX 04, France; patrick.sauvade@suez.com (P.S.); franz.coelho@suez.com (F.C.)

**Keywords:** ultrafiltration, air backwash, efficiency, viability of organic matter, shellfish hatchery effluents

## Abstract

Among all the techniques studied to overcome fouling generated in dead-end filtration, the injection of air during backwashes proved to be the most effective. Indeed, shear stress engendered by the two-phase flow enhanced particle removal on membrane surface. This work aims to study the injection of air to drain the membranes before backwash. Firstly, the efficiency of this backwash procedure was evaluated during the ultrafiltration of seawater on a semi industrial pilot plant using different operating conditions. Then, the treatment of seawater, doped with oyster gametes to simulate the filtration of shellfish hatchery effluents, was performed to confirm the hydraulic performance of the air backwash. Indeed, the release of gametes, expulsed by exotic bivalves in the natural environment, could be a risk for the biodiversity preservation. The impact of air backwash on the integrity of oocytes and spermatozoa was identified using flow cytometry and microscopic analyses. When oyster gametes were added, their retention by ultrafiltration was validated. The impact of air backwash on these species viability was a significant information point for the implementation of this process on shellfish production farms.

## 1. Introduction

Ultrafiltration (UF) is considered an efficient process to remove viruses, bacteria [1] and suspended matter to produce drinking water or to treat industrial effluents. In seawater applications, this process is widely used in pre-treatment for reverse osmosis (RO) desalination [2,3] or in aquaculture [4]. Although dead-end filtration requires no circulation, in contrast to cross-flow filtration [5,6], the accumulation of particles or colloids which result in a performance decrease is a more important consideration. Many improvements have been developed to overcome this phenomenon by manipulating membrane structure or by enhancing the efficiency of backwashing [7,8]. This procedure was studied using the filtration of natural water, with a combination of surface modifications of membranes [9] or for seawater treatment [10]. Backwash understanding and optimization was studied to link the key backwash parameters and characteristics of feed water [11,12,13,14,15,16,17,18,19]. Moreover, the influence of the type of water employed as backwash water was investigated [20,21], especially with regard to UF permeate versus ultrapure water. Optimization is based on the assessment of backwash efficiency under different operating conditions. The injection of air directly into the feed stream was also investigated. In the case of the treatment of clay suspensions by ultrafiltration, the filtration of this two phase flow showed an improvement of permeate flux [22]. Similarly, it was demonstrated that during wastewater treatment using submerged anaerobic membrane bioreactors, the biogas bubbles generated enhanced membrane fouling control [23]. The use of air during backwash is also an effective method to control the accumulation of matter on the membrane surface during dead end [18] or cross flow filtration [24]. Air assisted ultrafiltration of natural surface water, which consists of an injection of air during backwash, demonstrated that backwashes were more efficient with air injection to reduce fouling [25,26,27,28]. Indeed, two phase flow backwash is more efficient than classical backwash for membrane cleaning by increasing shear stress, which contributes to limiting the accumulation of particulate at the membrane surface. Combining a rinsing step and this injection of air during backwash led to a 65% energy savings for the filtration of natural water [29]. Reduced backwash times and use of chemicals are other advantages of air assisted backwashes. The conclusions are validated in the case of ultrafiltration for seawater pre-treatment before desalination. The dead-end ultrafiltration using air enhancement backwash was performed on Chinese seawater [30].

This work is part of a project that aims to study the membrane processes applied to hatcheries and nurseries in shellfish farming. Some hatcheries can produce exotic shellfish that introduce the riskof gametes, larvae and cells reaching the environment, threatening the local biodiversity. In this study, we focused on the air backwash efficiency for the treatment of these farming effluents. At first, the efficiency of air backwashes (AB) was evaluated on natural seawater in terms of hydraulic performances. Secondly, the effect of this AB was evaluated in the case of dead-end ultrafiltration of shellfish hatchery effluents. The AB performances were evaluated for four types of effluents. The impacts on hydraulic performances and membrane organic fouling viability, were demonstrated. 

## 2. Materials and Methods

### 2.1. Seawater and Effluents

The tests were carried out at the station Ifremer of Bouin (France), which was continuously supplied with natural seawater (Atlantic Ocean) from the Bay of Bourgneuf. These experiments were carried out over 6 months, from February to July 2017, taking into account the variability of the seawater for which the characteristics are presented in Table 1. Chlorophyll and pheo-pigment measurements were used to examine the degraded biomass. At the Ifremer station, seawater received several pretreatments: Settling in tailing ponds, filtration on sand filters (25–30 µm), and UV treatments. After these pre-treatments, only turbidity was analysed.

Effluents containing seawater with gametes, oocytes or spermatozoa, produced by oysters, *Crassostrea gigas*, were generated to simulate effluents that could be produced by a shellfish hatchery. Both effluents were prepared to simulate: (a) A chronic pollution and (b) an accidental pollution. The objective was to retain the entire population of gametes with: (a) low concentrated solutions of spermatozoa (spz) or oocytes (oo) for a period of 30 h, withthe targeted concentration in membrane entrance being 10,000 spz mL^−1^ or 69 oo mL^−1^; and (b) solutions with a higher concentration of gametes for a shorter time with the targeted concentrations being 200,000 spz mL^−1^ or 300 oo mL^−1^. 

### 2.2. Ultrafiltration: Membrane and Pilot Plant

All the experiments were conducted using SUEZ-aquasource^®^ hollow fibre membranes with an inside to outside configuration (Table 2). 

**Figure 1:**
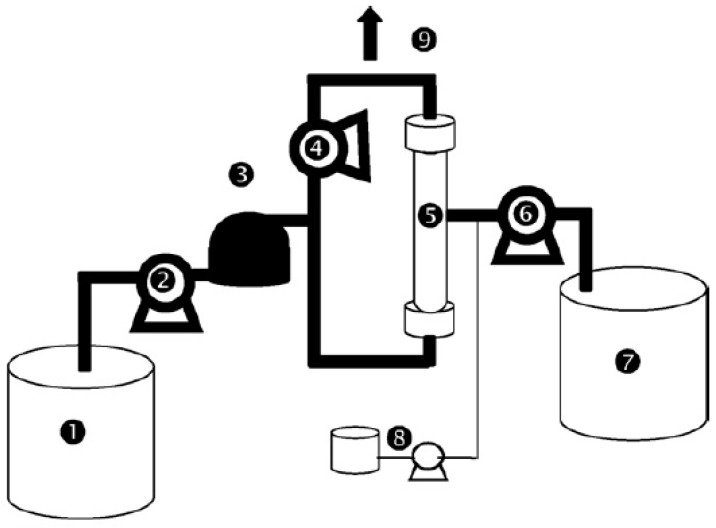
Pilot plant “image” (1—feed tank; 2—feed pump; 3—pre-filter; 4—recirculating pump; 5—membrane module; 6—backwashing pump; 7—purified water tank for backwash; 8—chemical cleaning part; 9—purge).

A semi industrial pilot plant (Figure 1 [31]), able to treat 20 m^3^ d^−1^, was used in this study. Completely automated, the process was able to adapt the filtration parameters to the quality of the input water. The ultrafiltration unit was equipped with a set of manual and automatic valves for the process. The automatic valves were pneumatic valves controlled by solenoid valves. The pressurized air was supplied by a compressor. To control the operating performances, pressure, temperature sensors, and flowmeters were connected to a data logger. These were then controlled by a programmable industrial. All the tests were carried out with dead-end filtration. Seawater continuously filled a 150 L tank and passed through a 130 µm prefilter before being pumped to the membranes. The permeate was collected in a 150-L permeate tank. 

### 2.3. Membrane Cleanings

To remove the fouling, different membrane cleanings were automatically performed by the pilot plant. These included classical backwashes (CB), air backwashes (AB) and chemical cleaning (CEB) (Table 3). CB consisted of an injection of permeate, at a flow rate of 2.5 m^3^ h^−1^, pumped from the permeate tank to the membrane with reverse flow. During AB, the water was discharged from the module. Hollow fibres were then filled with air until the pressure reached approximately 0.3 bar. This was immdiately followed by a backwashing operation. One AB was performed every 5 CBs. Using a limited permeability fixed at 300 L h^−1^ m^−2^ bar^−1^, a CEB was carried out. Firstly, permeate with an addition of sodium hydroxide (400 ppm, pH = 10) and sodium chlorine (150 ppm) was injected into the membrane. After 30 min of contact, an AB was performed. A mix of permeate and sulfuric acid (800 ppm, pH = 2) was then injected into the module for 30 min. A final AB was performed at the end of this CEB. Chemicals were injected using pilot-controlled dosing pumps. The chemicals (sodium hydroxide 30% EN896 (NaOH), sodium chloride 13% EN901 (NaCl) and sulfuric acid 37.5% EN899 (H_2_SO_4_)) used for the chemical cleanings of the membranes were purchased from Quaron (Rennes, France). Lp and TMP represent the membrane permeability and the transmembrane pressure, which were calculated and recorded continuously every minute. All the results accounted for the variation of the viscosity with the temperature.

### 2.4. Analyses

Flow cytometry is a fluorescence-based technique used on the suspensions of particles. The characteristics, size and shape of each cell are detected by scrolling through particles in laser beams. Oocytes and spermatozoa samples were analyzed on a Partec flow cytometer to determine their concentration at different points in the system (feed, inlet membrane, permeate, retentate) during the tests. Two 800 µL samples from each point were analyzed with a third measurement performed if necessary. Oocytes viability was evaluated using 2 fluorescence labels: SYBR-Green (which penetrates live and dead oocytes) and IP (which penetrates only cells with damaged membranes). 10 µL of SYB-Green and 5 µL of IP were mixed with 800 µL of the oocytes sample. This solution was then analyzed after 10 min in the dark [32]. Spermatozoa viability was evaluated using the same method but with SYBR-14 instead of SYBR-Green. Fluorescent labelling (SYBR-Green, SYBR-14, and IP) used for cytometry analyses was purchased from Fisher. In addition, oocytes and spermatozoa samples were observed on a phase contrast microscope connected to a video camera, with special attention given to their shape and cell integrity. Spermatozoa were filmed on a Malassez and cells were tallied to determine the rate of moving spermatozoa in the sample (movies were 5 s long). Moreover, pictures of oocytes were taken to characterize their status (shape) and the possible degradation of their membrane structure.

### 2.5. Operating Conditions

As for the industrial application, the ultrafiltration is operated at a constant permeate flux. The duration between each backflush is constant for each test. First, several seawater filtration tests were carried out with varying conditions of permeate flux, J, (60 to 100 L h^−1^ m^−2^) and filtration duration (20 to 60 min). For a period of 6 months, filtration parameters were monitored continuously to study the impact of fouling on hydraulic performances. Each test of flux/time of filtration was followed for a period of between 50–72 h until a sustainable flow was obtained [33]. To consider the variability of the composition of Atlantic Ocean water, each condition group (flux and time of filtration) was randomly reiterated twice during the 6-month period. A chemical cleaning of membranes was performed after every test and the initial permeability was recovered. Impact of CB and AB on filtration performances was determined for each condition. During a second step, the filtration of the seawater effluents containing gametes was performed. Two types of filtration were carried out for oocytes and for spermatozoa. The first was low concentrated effluents of spermatozoa or oocytes filtered for 30 h with a flux of 60 L h^−1^ m^−2^. Two conditions of time between backwashes were tested: 30 and 60 min [34]. Oocytes or spermatozoa at a low concentration were fed continuously into the feed tank supplied with seawater. The second stype of filtration invloved effluents with a higher concentration of spermatozoa or oocytes being treated for 5 h. The filtration conditions were a flux of 60 L h^−1^ m^−2^ with 60 min between the two backwashes. After each backwash (AB or CB), oocytes or spermatozoa with a high concentration were introduced by injection over a short period (<5 s) in the feed tank alimented with seawater.

## 3. Results

### 3.1. Seawater

**Figure 2:**
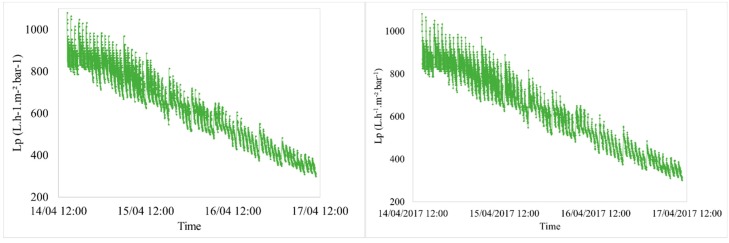
Variation of permeability vs. time (J = 60 L h^−1^ m^−2^ and t_filtration_ = 60 min for seawater filtration).

**Figure 3:**
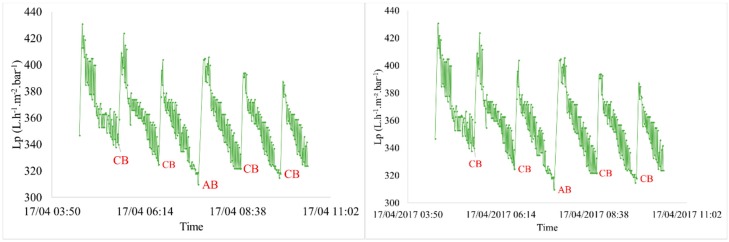
Variation of permeability vs. time (J = 60 L h^−1^ m^−2^ and t_filtration_ = 60 min for seawater filtration)—Zoom on a 7-hour period.

The filtration of pre-treated seawater from the Bay of Bourgneuf (France) was performed to compare the efficiency on membrane regeneration with two different backwashes. The evolution of permeability with time was similar in the different conditions tested. A noticeable decrease of permeability was observed with the filtration period (e.g., J = 60 L h^−1^ m^−2^ and t_filtration_ = 60 min (Figure 2 and Figure 3)). This decline is a function of two parameters: Quality of the feed water and the fixed filtration parameters. As the time of filtration, during which the permeate flux increases, there is more foulingwhich results in the permeability decreasing over time. To remove the fouling, the effect of AB efficiency compared to CB is highlighted (Figure 3). It shows that the evolution of permeability during six backwash zoom periods presents a better recovery after AB than after CB. 

**Figure 4:**
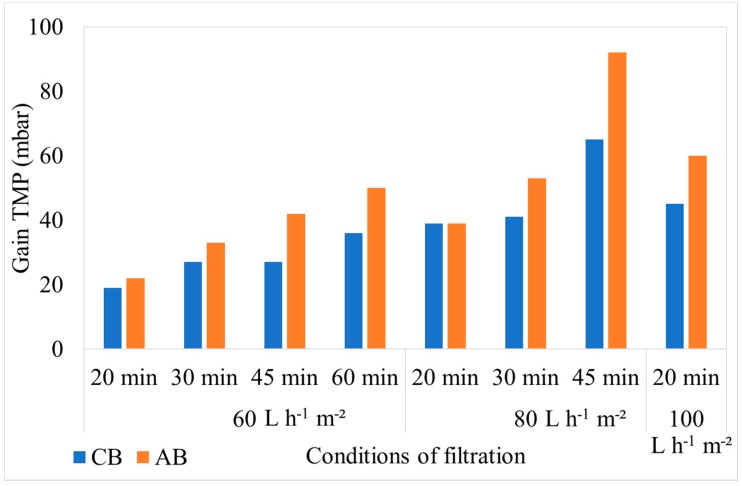
Gain of Transmembrane Pressure TPM after Classical Backwash (CB) and Air Backwash (AB)—filtration of natural seawater.

We can observe that the injection of air enhances the membrane cleaning because the rise of initial permeability is higher after AB than after the previous CB of the cycle. This assessment is confirmed whatever the couple (J; t_filtration_) of fixed conditions. After CB, a decrease of the initial permeability of the filtration cycle was still observed compared to the previous cycle, about 10 L h^−1^ m^−2^ bar^−1^; which is not the case after an AB. In order to estimate the enhancement of membrane cleaning due to the air injection, the benefit on TMP after backwashes was evaluated. The results obtained after an AB was compared to the average of the 5 previous results generated by CB. Figure 4 presents these results for different operating conditions. As the fouling increases, generated by an increase of driving force and/or filtration time, the TMP benefit builds up with or without air injection. However, whatever the operating conditions, the air injection increases the backwash efficiency (i.e., higher regeneration efficiency). For the same driving force (i.e., the same permeate flux) of 60 L h^−1^ m^−2^ and for different durations between 20 to 60 min, the regeneration of the membrane was improved from 15% to 39% by the injection of air. The longer the duration, the higher the improvement. Similar results were obtained for a constant duration and different permeate flux. These results lead to the conclusion that the efficiency of ABs remains significant for cleaning of membrane surfaces used for the filtration of seawater.

### 3.2. Shellfish Hatchery Effluent

#### 3.2.1. Hydraulic Effect

**Figure 5:**
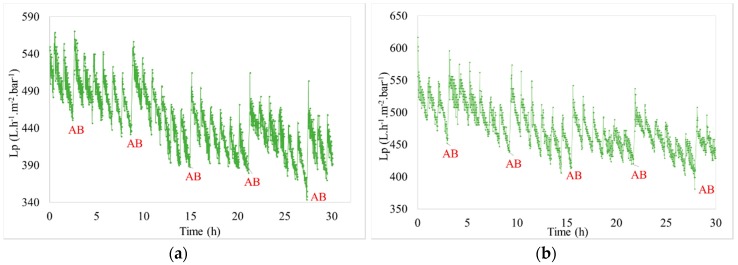
Variation of permeability vs. time (J = 60 L h^−1^ m^−2^ and t_filtration_ = 60 min for Chronic pollution)—(**a**) [oocytes]_feed_ = 69 oo mL^−1^ and (**b**) [spermatozoa]_feed_ = 10,000 spz mL^−1^.

**Figure 6:**
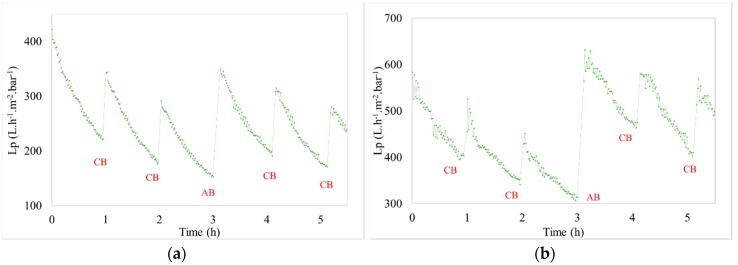
Variation of permeability vs. time (J = 60 L h^−1^ m^−2^ and t_filtration_ = 60 min; accidental pollution) (**a**) [oocytes]_feed_ = 300 oo mL^−1^ and (**b**) [spermatozoa]_feed_ = 200,000 spz mL^−1^.

The hydraulic efficiency was also evaluated for seawater effluent containing oyster gametes. The hydraulic performances were tracked during tests periods. For example, for chronic pollution (low concentrated effluents for 30 h), the evolution of permeability versus time, during the filtration of hatchery effluents containing oocytes or spermatozoa, are respectively presented by Figure 5a,b, and by Figure 6a,b for accidental pollution (high concentrated effluent for 5 h). 

**Figure 7:**
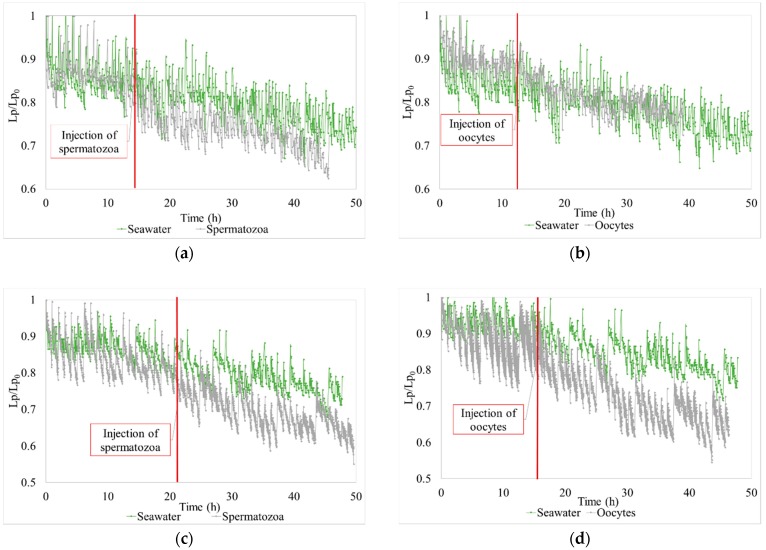
Variation of permeability vs. time during the 30 h filtration of gametes (chronic pollution). (**a**) Spermatozoa—[J = 60 L h^−1^ m^−2^ and t_filtration_ = 30 min]; (**b**) Oocytes—[J = 60 L h^−1^ m^−2^ and t_filtration_ = 30 min]; (**c**) Spermatozoa—[J = 60 L h^−1^ m^−2^ and t_filtration_ = 60 min]; (**d**) Oocytes—[J = 60 L h^−1^ m^−2^ and t_filtration_ = 60 min].

Due to the addition of oyster oocytes or spermatozoa, the fouling is higher than the fouling observed for seawater with the same operating conditions (Figure 7). The fouling or the TMP increases with a longer filtration time or a higher concentration in the effluent. Due to the difficulty in maintaining a constant high concentration (because of rapid and sequential injection of gametes for accidental pollution) two tests with similar filtration conditions were performed. For chronic pollution tests (low concentrated effluents during a long time), two tests were carried out with different filtration conditions.

**Figure 8:**
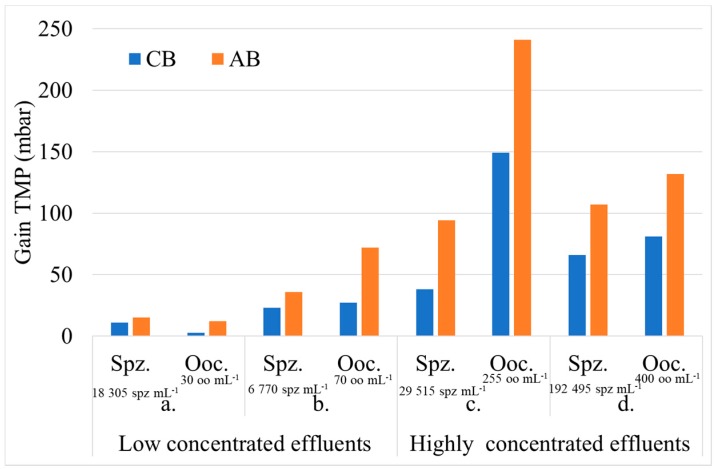
Gain TPM after CB and AB during the filtration of effluents. Chronic pollution (**a**) J = 60 L h^−1^ m^−2^ and t_filtration_ = 30 min and (**b**) J = 60 L h^−1^ m^−2^ and t_filtration_ = 60 min for similar feed concentration. Accidental pollution J = 60 L h^−1^ m^−2^ and t_filtration_ = 60 min for 2 feed concentrations (**c**,**d**).

In the case of the chronic pollution, the gain obtained with the air injection increases with the time of filtration. The improvement is between 329% and 167% for the oocytes and between 39% and 57% for the spermatozoa (Figure 8a,b). For accidental pollution, the gain of TMP is 65% and between 60% and 150% for oocytes and spermatozoa respectively. This was higher in the case of AB because air backwash improves the removal of gametes from membrane surfaces. These percentage values are a function of the type of gamete, the concentration injected, and the operating conditions. Air injection during backwashes clearly enhance regeneration and hydraulic performances whatever the operating conditions. 

#### 3.2.2. Impact on Biologic Matter Integrity

Flow cytometry and microscopic analyses were used to determine the number, the integrity and the viability of the gamete in the backwash effluent. Before treatment, the integrity measured in samples of effluent was greater than 80%, independent of the gametes and the concentration. Although it is not the objective of the present paper, no gamete is detected in the permeate regardless of the operating conditions (under detection limit, 350 spz mL^−1^). Therefore, ultrafiltration is clearly efficient enough to protect the natural environment from this type of effluent. In the case of the filtration of a chronic pollution and for two filtration durations, Table 4, Table 5, Table 6 and Table 7 present the flow cytometry data at different sampling points for oocytes or spermatozoa, for each day of each experiment.These take into account the effect on duration and an eventually variation of the inlet concentration. In the case of oocytes, the rate of oocytes degraded is presented. The spermatozoa or oocytes concentration measured at the membrane inlet is in agreement with the expected value. However, this concentration slightly fluctuates over time, and between the different experiments. This variation is explained by the dilution of gametes from a concentrated environment to the feed tank and the difficulty in maintaining a constant concentration value in the tank that supplied the UF membrane. The Volumetric Concentration Factor (VCF) presented in Table 4, Table 5, Table 6, Table 7, Table 8 and Table 9 was calculated as a function of the filtration time and the injected volume of permeate during backwash, with or without air. 

In the case of CB, as expected, gametes concentration in the backwash water was higher than in the feed solution. However, the VCF was not reached, reflecting that the backwash is not completely efficient. These results corroborate those obtained with one fiber [25] or for the filtration of seawater [35]. A mass balance comparison between filtration and backwash showed that some compounds were not removed at the end of the backwash. AB on this remaining fouling would have allowed a better membrane regeneration. With a volume of water almost identical to CB, it was expected that a much higher concentration of gametes would be found in AB water. However, the concentrations obtained were much lower, by a factor ranging from 1.15 to 7.3 depending on the operating conditions. The most important factors were discovered for the treatment of spermatozoa. Cytogram observations of these samples revealed the presence of a spermatozoa population outside the zone R1, where gametes are usually found. The hypothesis presented is an impairment of gametes during the AB. In fact, the membrane being dried when filled with air and the shear force induced when permeate water is injected during this process may have an impact on the physical integrity of these species. This assessment explains the low concentration of spermatozoa in AB water and the presence of another population of plots on the cytogram that doesn’t match with the characteristics of alive spermatozoa. In the case of oocytes filtration, the concentration of oocytes recovered in backwash water remained slightly lower with air injection. However, the test of integrity displays the impact of air injection on these species. It was found, according to this analysis, that about half of the oocytes were physically damaged during AB for every test performed as compared to CB. The difference in behavior between the spermatozoa and the oocytes could be explained by the fragility of the spermatozoa compared to the structure of oocytes. Experiments at high concentrations for accidental pollution (Table 8 and Table 9) confirm this drastic reduction of spermatozoa in the presence of air and the appearance of a different population on cytometry analyses suggest an impairment of the cells. In order to confirm these hypotheses, additional analyses using optical microscopy were carried out. In the case of the filtration of highly concentrated effluents, spermatozoa before treatment and in backwash water were filmed in order to characterize their mobility. The observation of three samples in the feed, in the CB and the AB supported the previous hypothesis. Even more spermatozoa were observed in AB water samples than in CB water samples (Table 10). None of them were moving when air was injected before the backwash, showing the impairment of the spermatozoa. 

**Figure 9:**
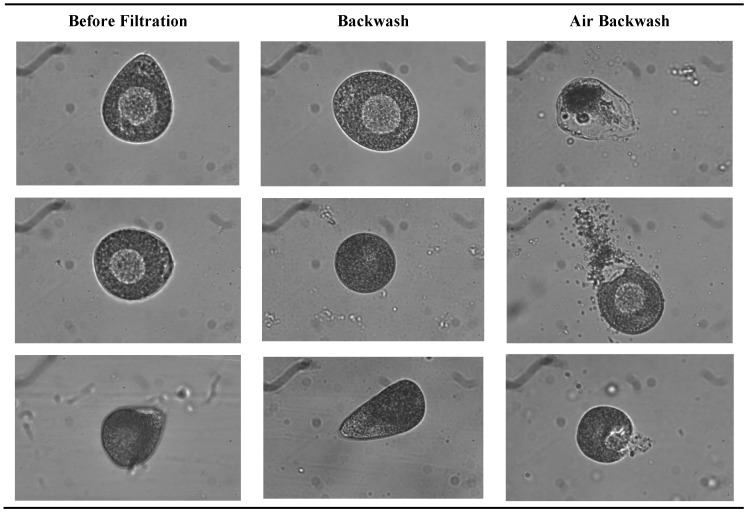
Pictures of oocytes before filtration and in backwash water.

As for the oocytes, pictures were taken to compare their shape and integrity before filtration and in backwash water. Samples of pictures taken in different waters are presented in Figure 9. The damaged oocytes pictures after AB waters were only observed in this effluent. The broken membrane structure of oocytes in these samples corroborated the previous analyses; oocytes are partially degraded by the injection of air before the backwash. Besides the impact on the efficiency of removing gametes from the membrane surface, ABs have another positive impact concerning the gametes integrity. This is true whatever the type of gamete treated, its concentration or the conditions of filtration tested. In order to secure shellfish production, this result would avoid the management and treatment of backwash water when air is injected beforehand, making ultrafiltration an attractive process for removing gametes in hatchery effluents.

## 4. Conclusions

The aim of the study was to compare the performances of AB to CB on membrane cleaning in the case of ultrafiltration of seawater effluents with or without oyster gametes. Dead-end ultrafiltration was performed in a hollow-fiber module. The air backwash was carried out in two consecutive steps: the module was first drained of water filled with compressed air until the pressure of 0.3 bar, then a CB was performed. The two-phase flow created, improved the membrane cleaning for the different types of effluent treated. The filtration of pre-treated seawater underlined the enhanced performance of AB in removing fouling compared to CB, whatever the operating conditions. The more the fouling increases, aided by the flow (driving force) and the filtration time (duration), the more the backwashes with air engendered a benefit to permeability, in comparison with the backwashes without air. These results confirm the benefits of AB as air injection greatly improves the removal of particulates leading to a reduction of cumulative fouling. The filtration of effluents containing gametes led to the same conclusion: although the fouling during these tests was more significant than in the filtration of seawater, because of the addition of spermatozoa or oocytes, the performance decrease was controlled thanks to ABs. The removal of organic matter deposited on the membrane surface was more efficient with air injection. This result was observed for different conditions of filtration and gametes concentrations in treated effluents. However, against all expectations, the concentration of spermatozoa obtained in AB water was lower than in a CB, and for oocytes the integrity of gametes obtained in AB water was lower than in a CB. It is the first time that a study showed the impact generated by ABs on hydraulic performances as well as on biologic fouling, and more especially on shellfish gamete integrity. The population of spermatozoa was strongly diminished in water from ABs compared to CB water. An impairment of these spermatozoa was observed with air contact, and the shear stress produced by the two-phases flow during ABs. The observation of spermatozoa mobility in these different waters and the flow cytometry analyses of AB water corroborate this hypothesis. In the case of oocytes filtrations, the flow cytometry analyses revealed a loss of integrity of these species during ABs whatever the conditions of treatment. Pictures of oocytes structures in these effluents validate this result. Gamete effluent is a waste that must be treated in the case of the production of exotic oysters, and ultrafiltration proves to be an excellent process with a retention greater than 3–4 Log (no the topic of this paper). The addition of air during backwashing increases hydraulic performance and degrades gametes integrity. The impact of AB on these species is significant information for the industrial development of this process. 

## Figures and Tables

**Table 1:** Natural seawater characteristics.

Characteristics	Min.	Max.	Average
Turbidity (NFU)	0.674	13.4	4.5
Salinity (%)	30.8	34.7	33.5
Chlorophyll a (µg L^−1^)	0.89	7.04	2.5
Pheo-pigments (µg L^−1^)	0.24	1.84	0.71

**Table 2:** Membrane characteristics.

Characteristics		
Material	Polyethersulfone	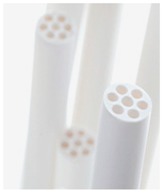
Inside diameter (mm)	0.9	
Molecular weight cut off (kDa)	200	
Numbers of channels	7	
Module area (m²)	8	
Water permeability (L h^−1^ m ^−2^ bar^−1^)	1000	

**Table 3:** Operating parameters of membrane cleanings.

Operating Parameters	CB	AB	CEB
Volume of permeate (L)	32	50	100
Time (min)	1	5	60
Frequency	20–60 min	5 CB	72 h

**Table 4:** Flow cytometry of spermatozoa—(J = 60 L h^−1^ m^−2^ and t_filtration_ = 30 min for chronic pollution).

Time	Membrane Inlet	Backwash	Air Backwash	Permeate
Day 1	30,705 spz mL^−1^	120,560 spz mL^−1^	8,345 spz mL^−1^	spz mL^−^^1^ < limit of detection
	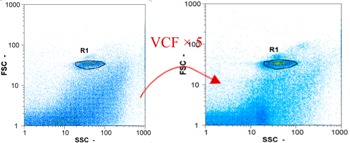		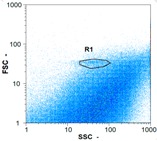	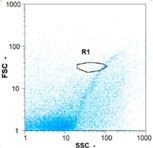
Day 2	18,305 spz mL^−1^	73,840 spz mL^−1^	10,170 spz mL^−1^	spz mL^−^^1^ < limit of detection
	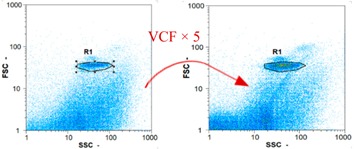		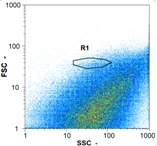	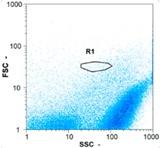

**Table 5:** Flow cytometry of oocytes—(J = 60 L h^−1^ m^−2^ and t_filtration_ = 30 min for chronic pollution).

Time	Membrane Inlet	Backwash	Air Backwash	Permeate
Day 1	45 oo mL^−1^ 16.67%	165 oo mL^−1^ 17.14%	185 oo mL^−1^ 62.50%	0 oo mL^−1^ -
	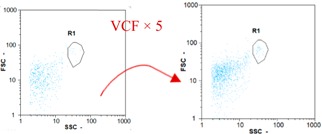		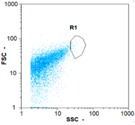	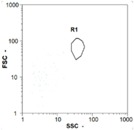
Day 2	30 oo mL^−1^ 14.29%	85 oo mL^−1^ 5.56%	30 oo mL^−1^ 33%	0 oo mL^−1^ -
	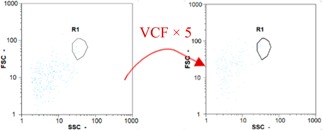		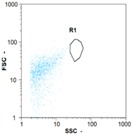	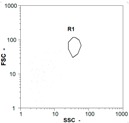

**Table 6:** Flow cytometry of spermatozoa—(J = 60 L h^−1^ m^−2^ and t_filtration_ = 60 min for chronic pollution).

Time	Membrane Inlet	Backwash	Air Backwash	Permeate
Day 1	5115 spz mL^−1^	22,485 spz mL^−1^	8100 spz mL^−1^	spz mL^−^^1^ < limit of detection
	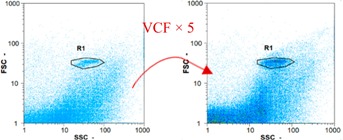		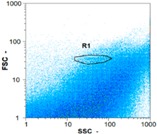	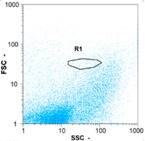
Day 2	6770 spz mL^−1^	53,850 spz mL^−1^	10,340 spz mL^−1^	spz mL^−^^1^ < limit of detection
	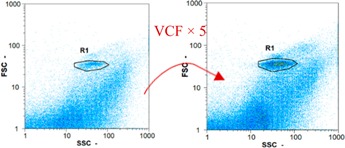		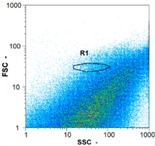	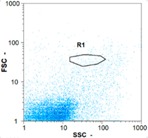

**Table 7:** Flow cytometry of oocytes—(J = 60 L h^−1^ m^−2^ and t_filtration_ = 60 min; chronic pollution).

Time	Membrane Inlet	Backwash	Air Backwash	Permeate
Day 1	55 oo mL^−1^ 0.0%	530 oo mL^−1^ 4.17%	390 oo mL^−1^ 5.32%	0 oo mL^−1^ -
	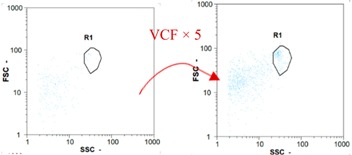		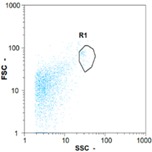	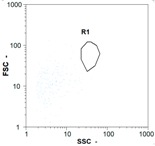
Day 2	70 oo mL^−1^ 5.56%	750 oo mL^−1^ 2.86%	570 oo mL^−1^ 19.18%	0 oo mL^−1^ -
	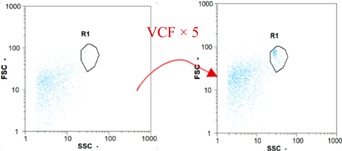		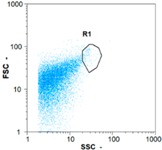	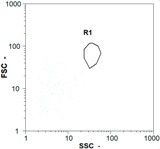

**Table 8:** Flow cytometry of spermatozoa—(J = 60 L h^−1^ m^−2^ and t_filtration_ = 60 min; accidental pollution) for 2 feed concentrations.

Test	Membrane Inlet	Backwash	Air Backwash	Permeate
Test 1	29,515 spz mL^−1^	121,600 spz mL^−1^	44,175 spz mL^−1^	spz mL^−^^1^ < limit of detection
	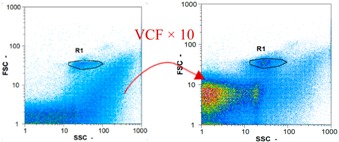		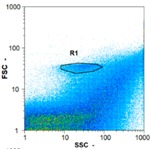	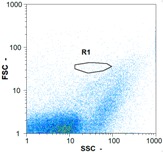
Test 2	192,495 spz mL^−1^	466,715 spz mL^−1^	11,560 spz mL^−1^	spz mL^−^^1^ < limit of detection
	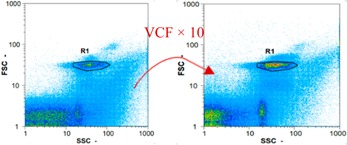		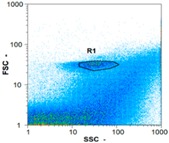	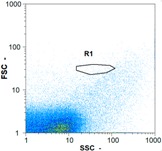

**Table 9:** Flow cytometry of oocytes—(J = 60 L h^−1^ m^−2^ and t_filtration_ = 60 min for accidental pollution) for 2 feed concentrations.

Test	Membrane Inlet	Backwash	Air Backwash	Permeate
Test 1	255 oo.mL^−1^ 8.06%	1430 oo.mL^−1^ 11.73%	1355 oo.mL^−1^ 48.47%	0 oo.mL^−1^ -
	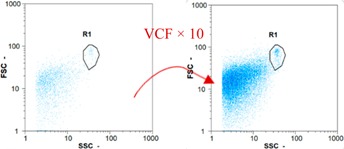		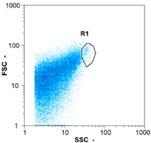	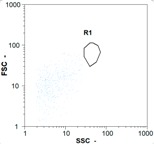
Test 2	400 oo.mL^−1^ 5.38%	2360 oo.mL^−1^ 6.14%	1535 oo.mL^−1^ 49.68%	0 oo.mL^−1^ -
	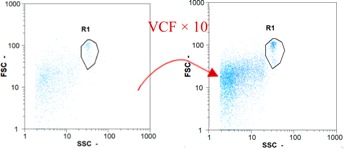		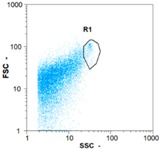	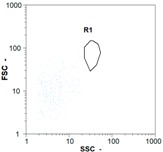

**Table 10:** Spermatozoa observations.

Water Sample	Sample	Number of Spermatozoa	Moving Spermatozoa (%)
Before filtration	1	79	19
	2	84	24
	3	84	15
CB	1	20	10
	2	61	8
	3	83	4
AB	1	108	0
	2	134	0
	3	98	0
